# Association of Methylenetetrahydrofolate Reductase (*MTHFR*) Polymorphism with Osteosarcoma in a Mexican Population

**DOI:** 10.3390/pediatric16030066

**Published:** 2024-09-09

**Authors:** Irma G. Enriquez-Maldonado, Daniel A. Montes-Galindo, Rocio Ortiz-Lopez, Jesus Ojeda-Ibarra, Margarita L. Martinez-Fierro, Iram P. Rodriguez-Sanchez, Augusto Rojas-Martinez, Angel Zavala-Pompa, Carmen Alicia Sanchez-Ramirez, Alejandra E. Hernandez-Rangel, Karmina Sanchez-Meza, Idalia Garza-Veloz, Alejandrina Rodriguez-Hernandez, Ivan Delgado-Enciso

**Affiliations:** 1 State Cancerology Institute of Colima, Health Services of the Mexican Social Security Institute for Welfare (IMSS-BIENESTAR), Colima 28085, Mexico; gabyenriquez75@gmail.com; 2 Faculty of Chemical Sciences, University of Colima, Coquimatlán 28400, Mexico; 3 Tecnologico de Monterrey, Escuela de Medicina y Ciencias de la Salud, Monterrey 64710, Mexicoaugusto.rojasmtz@tec.mx (A.R.-M.); 4 Department of Pathological Anatomy at Monterrey Specialty Hospital No. 25 (IMSS), Monterrey 64460, Mexico; 5 Academic Unit of Human Medicine and Health Sciences, Autonomous University of Zacatecas, Zacatecas 98160, Mexico; margaritamf@uaz.edu.mx (M.L.M.-F.); idaliagv@uaz.edu.mx (I.G.-V.); 6 Molecular and Structural Physiology Laboratory, School of Biological Sciences, Autonomous University of Nuevo Leon, San Nicolás de los Garza 66455, Mexico; 7 School of Medicine, University of Colima, Colima 28040, Mexicoarodrig@ucol.mx (A.R.-H.); 8 Robert Stempel College of Public Health and Social Work, Florida International University, Miami, FL 33199, USA

**Keywords:** methylenetetrahydrofolate reductase, osteosarcoma, folic acid, Mexico, single-nucleotide polymorphism

## Abstract

The methylenetetrahydrofolate reductase (*MTHFR*) gene 677C➔T polymorphism is capable of altering folate metabolism and can modify certain neoplasia risk. Reports have suggested that folate can have an influence on bone development and so it is of interest to know if the *MTHFR* 677C➔T polymorphism is associated with the malignant transformation process of this tissue. The polymorphism was determined in 55 patients with osteosarcoma and in 180 healthy individuals. Compared with C/T+C/C genotypes, a 3.7-fold reduction in osteosarcoma probability is possible with the T/T genotype (OR 0.27, CI 95% 0.07–0.82). Undoubtedly, further studies, utilizing large samples and carried out on different populations, are necessary to confirm these results.

## 1. Introduction

Folate, a key mediator in the transfer of one-carbon units essential for methylation and nucleotide biosynthesis, plays a crucial role in DNA stability, synthesis, repair, and methylation. There is robust evidence demonstrating that folate has beneficial effects on health outcomes, including mortality, metabolic conditions, cardiovascular diseases (CVDs), neurocognitive disorders, cancers, and various birth outcomes. This body of evidence supports current guidelines advocating for daily folate supplementation, although its precise role in some diseases remains uncertain or debated. The health benefits of folate are thought to arise from several mechanisms. First, folate acts as a cofactor for methionine synthase, an enzyme that facilitates the conversion of homocysteine, thereby establishing an inverse relationship between folate levels and homocysteine levels. Elevated homocysteine (hyperhomocysteinemia) has been associated with higher risks of adverse pregnancy outcomes, cancers, CVDs, and neurological conditions. Second, variations in the 5,10-methylenetetrahydrofolate reductase (MTHFR) enzyme, which directs folate metabolites towards DNA methylation or synthesis pathways, may influence an individual’s susceptibility to certain conditions [1].

Folate metabolism has been shown to have an influence on bone growth [2]. Iqbal et al. showed that the prolonged administration of the antifolate agent, methotrexate (MTX), can suppress bone growth in young mice and that folate administration produces the opposite effect [3]. MTX administered in high doses is one of the therapeutic components used to fight uncontrolled malignant cell growth in bone tissue [4]. In epidemiological studies, folate has been associated with bone formation [5]. Women with osteoporosis have lower serum folate levels, and bone mineral density (BMD) progressively increases as serum folate levels increase [6]. Although complex molecular mechanisms are involved in the proliferation, differentiation, and apoptosis of bone cells, there is also evidence that folate metabolism could be involved in these processes [7,8]. However, it is not yet known if this metabolism could also have an influence on the malignant transformation of bone tissue. The most common primary bone cancer is osteosarcoma (OS), which is characterized by the proliferation of cells that directly produce osteoid [4]. Osteosarcoma has the potential to manifest in any bone throughout the body, although it typically originates in the metaphysis of long bones. While commonly recognized as a sarcoma affecting children, its occurrence follows a two-phase pattern across age groups, with an initial peak during childhood and adolescence, followed by a secondary peak in individuals aged 65 and older [7].

Folate ingestion has been shown to modify certain neoplasia risk, although the mechanism by which this is achieved is not yet clearly understood [9]. Chronic folate deficiency may cause alterations in DNA synthesis or repair, as well as changes in its methylation [9,10]. This could have a role in oncogene activation and in tumor suppressor gene inactivation [10]. However, folate bioavailability, or that of its metabolites, is not solely determined by ingestion, due to the fact that different enzymes participate in its metabolism. One such conspicuous enzyme is MTHFR. It catalyzes the synthesis of 5-methyltetrahydrofolate, the principal form of circulating folate, a carbon donor for homocysteine to methionine remethylation and a precursor of S-adenosylmethionine [11]. 

S-adenosylmethionine is the methyl group universal donor for methylation reactions, including that of DNA [12]. In 1995, Frostt et al. found a gene variant (polymorphism) in the 677 position (T instead of C) of the *MTHFR* gene. Whether in its 677T/T homocygote state or its 677C/T heterozygote state, the enzyme activity of this variant is significantly reduced [13]. Individuals with the 677T/T genotype have demonstrated DNA hypomethylation, which may be one of the molecular events taking place in the early stages of carcinogenesis [14]. 

The 677T variant has been controversially related to different neoplasias. It has been associated with possible greater risk for breast [15], esophageal [16], and stomach cancer [17,18] while at the same time acting as a protective factor against malignant lymphomas [19] and some types of leukemia [20,21]. It was reported in Turkish and Spanish populations that the 677T variant was not associated with OS development [22,23]. However, the association of the same genetic polymorphism with a given disease can be different, depending on the population studied, due to variations in population backgrounds and environments. The 677T variant is not very frequent in Turkey [22]. In contrast, its frequency is one of the highest in the world in Mexico [24]. It is therefore of interest to analyze whether the genetic alteration that affects folate metabolism could have an influence on the malignant transformation of bone tissue in the Mexican population. The present study was carried out as a case–control study to determine if there was an association between the *MTHFR* 677 polymorphism and OS in a northeastern Mexican population. 

## 2. Materials and Methods

Paraffin-embedded tumor tissue biopsies were obtained from 60 patients diagnosed with OS in the Pathology Department of the Hospital de Especialidades N° 25 del IMSS, in Monterrey, NL, Mexico. Peripheral blood was collected from 180 clinically healthy volunteers for comparison purposes at hospitals in the same city. OS patients and the control group were unrelated Mexican mestizo subjects from northeastern Mexico. The study was approved by the Committee on Ethics of the University of Colima’s School of Medicine, Mexico, authorized by the Department of Education of the Hospital de Especialidades N° 25 del IMSS NL, Mexico, and carried out in compliance with the Declaration of Helsinki. Participants in the control group (clinically healthy volunteers) signed an informed consent form. The Ethics Committee waived the need for informed consent from the patients in the case group, since samples obtained retrospectively and stored in the Department of Pathological Anatomy were analyzed from them, in addition to their clinical data being obtained from the clinical record. The above does not represent a risk for these subjects, guaranteeing anonymity and the protection of personal data. The above was in accordance with the functions provided to the Ethics Committees by the General Health Law on Health Research in Mexico, being a risk-free investigation [25]. DNA was extracted from the paraffin-embedded tissue [26] and peripheral blood [27], using previously described techniques. *MTHFR* gene 677 polymorphism determination was carried out by PCR restriction fragment length polymorphism (PCR-RFLP) [12]. Ten percent of samples were analyzed using a different genotyping method, namely, allele specific oligonucleotide hybridization [28], and identical results were obtained. 

The association between polymorphism and OS was carried out by means of odds ratio (OR) determination with its confidence interval (CI) and the χ^2^ test, using the EpiInfo 2000 computerized program [29]. The Hardy–Weinberg equilibrium of the genotypic distribution was calculated using the Online HW computerized program [30]. Statistical significance was considered when *p* < 0.05. 

The datasets used and/or analyzed during the current study are available from the corresponding author on reasonable request.

## 3. Results

Twenty-four of the OS patients were women and thirty-six were men, with a mean age of 18 years (8–67-year range). Age and sex differences between the control and OS groups were not shown. OS histological varieties were osteoblastic (53.3%), condroblastic, fibroblastic, telangiectasic (15% each), and small-cell (1.7%). OS was more frequently localized in the distal third of the femur (31.6%), followed by the proximal third of the tibia (21.6%), proximal third of the humerus (16.6%), inferior maxillary bone (11.6%), pelvis (3.3%), femoral diaphysis (3.3%), paranasal sinuses, clavicle, rib, proximal third of the femur, distal third of the tibia, astragalus, and soft tissues of the scapular-humeral region (1.7% in each of the latter locations).

DNA was extracted from the 60 paraffin-embedded OS biopsies and the *MTHFR* polymorphism was determined in 55 of them and in 180 controls (there was not a sufficient quantity of DNA obtained in 5 of the OS samples). The C/C, C/T, and T/T polymorphism percentages in the OS group were 32.7, 60, and 7.3%, respectively, and 26.7, 51.1, and 22.2% in the control group. Genotype distribution was in accordance with the Hardy–Weinberg equilibrium in the control group but was out of balance in the OS group (*p* = 0.048). As shown in Table 1, the 677T/T genotype was significantly less likely to be present in OS patients (7.3%) compared with control subjects (22.2%) (*p* = 0.02), whereas the presence of a single 677T allele (heterozygote) was not different between groups (60% vs. 51.1%, in cases and controls, respectively; *p* = 0.89).

## 4. Discussion

The present work indicates that the *MTHFR* 677TT genotype was significantly less likely to be present in OS patients (7.3%) compared with control subjects (22.2%) (*p* = 0.02), in a Mexican population. Various reports have suggested that folate metabolism may have an influence on bone development. Folate ingestion during pregnancy has a positive effect on fetal BMD [31,32]. Folate levels in blood have the same positive BMD effect in post-menopausal women [33,34,35] 

On the other hand, a deficiency in folate metabolism, brought on by the administration of anti-folate agent MTX, has been shown to possibly suppress bone growth in mice [3]. On the genetic level, the *MTHFR* 677T/T genotype has a negative effect on folate metabolism causing reduced MTHFR activity [36]. It catalyzes the synthesis of 5-methyltetrahydrofolate, the principal form of circulating folate, a carbon donor for homocysteine to methionine remethylation and a precursor of S-adenosylmethionine. S-adenosylmethionine is the methyl group universal donor for methylation reactions, including that of DNA [27]. The *MTHFR* 677T/T genotype has been associated with BMD decrease [37] and fractures due to osteoporosis [38], suggesting that this genotype, as well as folate levels, are capable of having an influence on bone metabolism. *MTHFR* C677T might have some relationship with liver toxicity and fever after MTX therapy [39]. It has been reported that in pediatric osteosarcoma patients, the *MTHFR* 667TT polymorphism is associated with a higher relapse rate, although it does not predict overall survival. Therefore, identifying the *MTHFR* genotype could serve as an important predictor for assessing the likelihood of relapse and may be useful for risk stratification in future treatment protocols. Patients with this genotype may exhibit reduced sensitivity to Methotrexate treatment, suggesting that alternative chemotherapeutic agents could be more effective [40]. However, further studies on this topic would be necessary to implement it as a marker to guide treatment.

The present work indicates that the *MTHFR* 677T/T genotype probably produces a 3.7-fold OS risk reduction in a Mexican population. The above-mentioned information suggests that TT genotype was significantly less frequent in the OS group. Seen from a different perspective, it could be said that CC genotype was more frequent in OS (Table 1). The probable protection of the TT genotype against OS development could be the result of a negative effect of the mutant genotype on the accelerated and uncontrolled growth of neoplastic bone cells. However, this hypothesis still needs to be proved. 

Ozger et al. (2008) and Patiño-Garcia et al. (2009) reported that the *MTHFR* 677 polymorphism may not be an important factor in OS development in Turkish and Spanish populations [22,23]. Although they found no association between *MTHFR* genotypes and OS, they observed that the *MTHFR* 677C allele was more frequent in the OS group. These findings partially align with the results of the present study, suggesting the need for further research to confirm the role of *MTHFR* 677 polymorphism genotypes in osteosarcoma. The influence of *MTHFR* 677 polymorphism genotypes varies across different neoplasms, ethnic groups, and methylation patterns, complicating our understanding of its role in carcinogenesis [41,42]. This complexity is further exacerbated by interactions with environmental factors [24], including nutritional influences [43,44].

In the Mexican population, the 677TT genotype has previously been identified as reducing the likelihood of developing cervical cancer, but only in women with other risk factors such as multiple gestations and an early onset of sexual activity [24]. The variability in the influence of *MTHFR* 677 genotypes across different neoplasms and populations is evident. For example, the T allele is associated with a decreased risk of prostate cancer in the Asian population, but not in other populations [45]. Conversely, this same allele has been linked to an increased risk of bladder cancer [42], a finding that is also observed in ovarian cancer, but only in the Asian population [41]. In breast cancer, the role of the alleles of this polymorphism varies depending on the studied population [46], while in gastric cancer, it has been concluded that the genotypes of this polymorphism are not associated with the disease [47]. In acute lymphoblastic leukemia (ALL), the most common childhood cancer, the CC and CC + CT genotypes have been found to increase the risk of ALL in Asian children, with the TT genotype being significantly less common among ALL patients. However, this relationship was not observed in Caucasians [48]. This important variability between populations and neoplasms may reasonably explain the lack of complete concordance between previous reports analyzing this polymorphism in OS and the present study.

The lower proportion of the 677T/T genotype in patients with OS is congruent with reports that have suggested that MTX and the 677T/T genotype may have a negative influence on bone growth, at least when it is neoplastic. A protective factor of the 677T/T genotype against neoplasia development has been reported for certain types of leukemia [18,20], cancer of the bladder [49,50], and in some reports of colon cancer [51]. However, the mechanism by which this genotype helps protect individuals from neoplasia is not clear. It has been proposed that the reduced activity of the MTHFR enzyme causes a less efficient transformation of 5, 10, methylenetetrahydrofolate to 5 methylenetetrahydrofolate. This causes alterations in the homocysteine cycle but brings with it an intracellular accumulation of folate metabolites that take part in the process of DNA synthesis, which could confer protection [21]. The 677T/T genotype has also been associated with changes in DNA methylation (DNA hypomethylation in general and hypermethylation in some genes) [14], and multiple changes at this level have been detected in OS [23,52]. This suggests that the association between the *MTHFR* 677 polymorphism and OS could be partly caused by methylation patterns which are favored or limited by the presence of *MTHFR* genotypes. 

A relationship has been found between the 677T/T genotype and methotrexate toxicity in osteosarcoma patients [53,54,55]. Despite the fact that this is a very interesting subject, the association between the *MTHFR* genotype and the response to chemotherapy or toxicity has had contradictory results in other malignancies, such as hematological cancers [56,57]. Other functional polymorphisms in the same gene (such as 1298A/C) or in different genes of the folate pathway could also be analyzed in further studies. 

The lack of Hardy–Weinberg equilibrium (HWE) in *MTHFR* 677 genotype distribution observed only in the OS group could be a confirmation of a true association between this polymorphism and the disease [58], although the small sample size (n = 55) cannot be ruled out as the reason for this lack of equilibrium. Genotyping errors, an important cause of deviation from HWE, were not detected by a second genotyping methodology used in this study. However, bias may have been introduced into the study by the different DNA sources (blood for the controls and paraffin-embedded tissue for the cases). Another study limitation was the absence of environmental factors, such as diet. It has been demonstrated that polymorphisms can interact with multiple factors to modify neoplasia risk and therefore environmental factor analysis should be included in future studies on *MTHFR* and OS. 

Differences in tumor manifestation, the potential to undergo effective chemotherapy, and variations in the fundamental biology of tumors, are aspects to consider between pediatric and adult patients with osteosarcoma [7]. An important characteristic of the present study was that it included osteosarcoma patients between 8 and 67 years old (mean age of 18 years). This must be taken into account when comparing the present study with similar studies. 

## 5. Conclusions

In conclusion, it can be stated that the *MTHFR* gene 677T/T genotype is negatively associated with OS in the population studied, occurring in a significantly lower proportion in patients with OS than in healthy subjects. This suggests that the folate mechanism is a factor that may have an influence on the malignant transformation of bone cells. Undoubtedly, further studies using large samples and carried out on different populations are necessary to evaluate the role of the *MTHFR* polymorphism in OS development. 

## Figures and Tables

**Table 1:** Comparison of the *MTHFR* gene 677T/T genotype with other genotypes, between cases (patients with osteosarcoma) and controls.

Genotypes	Cases *n* = 55	Controls *n* = 180	OR (95%CI)	*p* *
C/C	18	48	Reference	
T/T ‡	4	40	0.27 (0.06–0.91)	0.03
C/T	33	92	Reference	
T/T ‡	4	40	0.28 (0.07–0.87)	0.02
C/C-C/T	51	140	Reference	
T/T ‡	4	40	0.27 (0.07–0.82)	0.02
C/C	18	48	Reference	
C/T ‡	33	92	0.96 (0.46–1.98)	0.89

An association analysis was performed to obtain the crude odds ratios (OR) with their 95% confidence intervals (CI) and *p* values. The genotypes considered as exposure factor (‡), or as reference, are indicated. * Chi-square test with Yates correction.

## Data Availability

The datasets used and/or analyzed during the current study are available from the corresponding author on reasonable request.
